# Wavelet Based Method for Congestive Heart Failure Recognition by Three Confirmation Functions

**DOI:** 10.1155/2016/7359516

**Published:** 2016-02-02

**Authors:** K. Daqrouq, A. Dobaie

**Affiliations:** Electrical and Computer Engineering Department, King Abdulaziz University, P.O. Box 80230, Jeddah 21589, Saudi Arabia

## Abstract

An investigation of the electrocardiogram (ECG) signals and arrhythmia characterization by wavelet energy is proposed. This study employs a wavelet based feature extraction method for congestive heart failure (CHF) obtained from the percentage energy (PE) of terminal wavelet packet transform (WPT) subsignals. In addition, the average framing percentage energy (AFE) technique is proposed, termed WAFE. A new classification method is introduced by three confirmation functions. The confirmation methods are based on three concepts: percentage root mean square difference error (PRD), logarithmic difference signal ratio (LDSR), and correlation coefficient (CC). The proposed method showed to be a potential effective discriminator in recognizing such clinical syndrome. ECG signals taken from MIT-BIH arrhythmia dataset and other databases are utilized to analyze different arrhythmias and normal ECGs. Several known methods were studied for comparison. The best recognition rate selection obtained was for WAFE. The recognition performance was accomplished as 92.60% accurate. The Receiver Operating Characteristic curve as a common tool for evaluating the diagnostic accuracy was illustrated, which indicated that the tests are reliable. The performance of the presented system was investigated in additive white Gaussian noise (AWGN) environment, where the recognition rate was 81.48% for 5 dB.

## 1. Introduction

The electrical activity signal of the heart's work, termed electrocardiogram (ECG), is recording of the electrical signal generated by the heart muscle during the cardiac cycle. The ECG signal is used diagnostically by cardiologists for pursuing the heart syndromes. The main challenge in heart disease diagnosis by means of ECG is that the normal ECG for each person might be totally different; sometimes, one disease has dissimilar signs on different patients ECG signals. Furthermore, two dissimilar syndromes could have, somehow, the same effects on ECG signals form. These dilemmas make the heart disease detection very hard. Therefore, the using of pattern classifier techniques can improve the ECG arrhythmia diagnoses [1–4].

Congestive heart failure is a serious clinical syndrome that comes from the advanced process of heart remodeling, in which mechanical and biochemical forces modify the shape, size, and functionality of the ventricle's ability to pump sufficient oxygenated blood. Compensatory process of regulated heart rate (HR), vasoconstriction, and hypertrophy eventually fail, leading to the distinguishing syndrome of heart failure: decreased cardiac output, sodium and water retention, elevated ventricular or atrial pressure, and circulatory and pulmonary congestion diagnoses [5].

Arrhythmia is a common clinical term for any cardiac rhythm that diverges from a normal ECG known as normal sinus rhythm. Arrhythmia is not considered in all cases as an irregular heart behavior [5] like in case of respiratory sinus arrhythmia, which is a natural periodic variation that occurs in *RR* intervals, corresponding to normal respiratory mechanism [6]. The heart rate, normal, slow, or fast, impulse formation may originate in pace-making cells in the sinoatrial (SA) node or ectopically [7, 8]. So, the finding of abnormal cardiac rhythms and automatic classification of the normal heart activity became a crucial task for clinical motives. The literature reports the detection and identification of life-threatening arrhythmias and, particularly, congestive heart failure, ventricular and atrial fibrillation, and ventricular tachycardia. Several detection algorithms have been suggested, such as the sequential hypothesis testing [9], the multiway sequential hypothesis testing [10], the threshold-crossing intervals, the autocorrelation function, the VF filter [11], and neural networks based algorithms [12]. Time-frequency (*t*-*f*) analysis [13] and wavelet analysis [14] have also been utilized. Current approaches utilize complexity measure [15] and multifractal investigation joined with a fuzzy Kohonen neural network [16, 17]. In the literature, several time domain and frequency domain methods had been employed to measure heart rate variability (HRV) for recognizing normal and CHF signals at different segment lengths [18]. The wavelet transform is one of the attractive tools used [18], where the standard deviation of the normal cases shows greater fluctuations than those exhibiting heart failure arrhythmias. It was probable to totally distinguish between 12 CHF and 12 NSR cases. Some of the time domain measures, such as the standard deviation of the averages of certain intervals in all 5 min segments and the standard deviation of the normal *R*-*R* interval, correlated significantly with severity (EF) of heart failure [19]. Maximum accuracy of 93.2% was also reached by [19] in separating 52 normal rhythms cases and 22 CHF patients using linear discrimination analysis. For arrhythmias, previous works mostly used time-frequency analysis techniques, statistical tools, and sequential analysis methods. Wigner Ville distribution technique and Choi-Williams distribution were employed for the short-term and long-term time-frequency analysis. Many works observed that many arrhythmias have time-varying characteristics [20, 21]. Güler et al. proposed an ECG beat classifier using the PhysioBank database and a combined artificial neural network (ANN) model, with higher accuracy of 97% when compared to the use of stand-alone neural network model [22, 23]. ANN is a famous classifier that may be used for ECG arrhythmia classification [24]. Multilayer perceptron (MLP) is used to classify ECG signals more accurately compared to other ANN methods. Still, MLP, especially with backpropagation training, suffers from slow convergence to local and global minima [25]. Progress of ANNs performance has been the subject of interesting research on ECG arrhythmia classification by using various feature extraction techniques. Özbay et al. compared the competence of fuzzy clustering neural network architecture with multilayered perceptron with a backpropagation training algorithm for classification of arrhythmias. The study proved the superiority of the presented system in terms of classification time, which is a result of decreasing the number of segments by grouping similar segments in training data with fuzzy c-means clustering [23]. A discrete wavelet transform is used to improve the quality of MLP with (BP) a training algorithm and also compared with other feature extraction algorithms and data reduction methods [26]. Many researchers have combined the MLP neural network with DWT for better accuracy [27]. Besides, an ECG beat classification system based on DWT and a probabilistic neural network (PNN) is proposed to differentiate six ECG beat types [28]. The ECG recordings were treated by means of CWT and DWT in an effort to predict the maintenance of sinus rhythm after cardioversion in patients with detected atrial fibrillation [2, 29]. Several classical methods of system analysis have been used in a morphological classification of the P-wave. Both ANN [30] and system modelling [31] have been shown to be superior to conventional frequency domain and signal-averaged ECG methods (achieving an accuracy of about 85%). Researchers have found good reasons for examining the effectiveness of a wavelet-linear discriminant analysis P-wave classification system [32]. While the utilization of wavelets classification of biomedical signals, including some components of the ECG, is well known [33, 34], wavelet analysis specifically of the P-wave has not received much studying. Secondly, neural networks functionally different from linear discriminant analysis (i.e., those with a hidden layer) require large samples because of the huge number of parameters to be extracted. Often, this is not practical. Third, linear discriminant analysis is relatively assumption-free, unlike model-based approaches [32]. We investigated some ECG signal processing problems in previous studies. For example, ECG arrhythmias, particularly tachycardia and bradycardia, were studied by DWT and the standard deviation was calculated over a particular DWT subsignal to classify the arrhythmias by means of the calculated parameters [35]. For enhancement, the particular wavelet functions were utilized to filtrate the ECG in high-pass band subsignals, as well as low-pass band subsignals [36]. Another work also investigated the quality of the reconstructed ECG signal of the data compression algorithms by calculating a collection of objective measures over DWT subsignals [37]. In addition, an investigation of the threshold that is suitable for ECG signal denoising was conducted. A wavelet transform threshold that is suitable for denoising of this type of biomedical and nonstationary signal was proposed, and the results were compared with Alfaouri and Daqrouq's threshold [38]. The mentioned publications commonly used the tool of DWT, although in each study different approaches were also suggested. Discrete wavelet transform has attracted a great deal of attention in the last two decades and has proved beneficial for immense nonstationary signal dilemmas. However, WPT has gradually caught researchers' attraction. The reason is the capability of the signal processing over the two types of WT coefficients: high-pass subsignal coefficients and low-pass subsignal coefficients. The presented work investigates the classification of CHF signals by WPT. The energy of certain subsignals is used for feature extraction. For the classification, three confirmation methods are suggested. In this paper, the CHF recognition system is studied in the context of recognition rate. This work studies several methods for improving the proposed work. Our purpose is to improve the performance of the WPE technique's utility in several types of arrhythmias. For this reason, many published techniques are investigated. The structure of this paper is as follows: firstly, the wavelet packet transform feature extraction method is presented, followed by a classification technique. Next, results and discussion will be presented, followed by the conclusion.

## 2. Method

### 2.1. Theoretical Overview of Wavelet Packet

The wavelet packet decomposition is a representation that offers a much better signal analysis. Wavelet packet atoms are indexed by three crucial parameters: position, scale as in wavelet transform decomposition, and frequency. Subsequently, the wavelet transform is presented as the inner product of a signal *x*(*t*) with the mother wavelet *ψ*(*t*):

(1) ψa,bt=ψt−baWψxa,b=1a∫−∞+∞xt∗ψt−badt,

where *a* and *b* are the scale and shift parameters. The mother wavelet is dilated or translated by *a* and *b*. Fundamentally, the WPT is very similar to DWT but the WPT decomposes both details and approximations instead of only performing the decomposition process on approximations. The pair of low-pass and high-pass filters in WPT are used to achieve the sequences to obtain different frequency subband features of the original signal. The two wavelet bases obtained from a previous node are defined as

(2) ψj+12pk=∑n=−∞∞hnψjpk−2nψj+12pk=∑n=−∞∞gnψjpk−2n,

where *h*[*n*] and *g*[*n*] denote the low-pass and high-pass filters, respectively. In (2), *ψ* is the wavelet function and *j* and *p* are the number of WPT levels and nodes of the previous node, respectively [39].

Wavelet transform is a very attractive method for arrhythmias analysis, particularly when we are dealing with arrhythmia that exhibits a change in the frequency, which, generally speaking, is very common. The fact that the signal can be decomposed into different wavelet subsignals of different band passes of frequency makes wavelet transform immensely useful in separating the arrhythmias frequencies in a given object. Therefore, the detection of the arrhythmia in related subsignals can be achieved easily.

### 2.2. Wavelet Packet Using for Feature Extraction Method

The wavelet packet is used to extract additional features for a higher classification rate [40]. In this study, WPT is applied for ECG feature extracting. Generally speaking, these data are not suitable for classification due to the huge length of the resulting data. Thus, we have to find out additional representation of the ECG features. A method to calculate the entropy value of the wavelet norm in digital modulation recognition was proposed [41]. In [42], a combination of the genetic algorithm and wavelet packet transform used for pathological evaluation was presented. The original ECG was decomposed in a set of discrete packet wavelets that transformed coefficients with different temporal and spectral features [43], showing that it is possible to obtain atrial activity with a finite set of these blocks and the inverse transform. In [44], the application of wavelet packet transform for atrial fibrillation was suggested. The nonterminating and the short-time terminating AF were successfully differentiated via the difference of log-energy entropies of two types of AF. In this paper, we use the percentages of energy obtained from the terminal nodes of the WP tree for CHF arrhythmias feature vector construction (from an ECG) to be used for diagnosing [1]. The proposed feature extraction method is summarized as follows:

(i) Preprocessing and normalization: prior to the stage of feature extraction, the ECG data are preprocessed and normalized to remove prospective fluctuations of baseline, interferences, noises, and so forth [1].
(ii) WP tree decomposing: the ECG signal is decomposed into WP at level five. Then, we propose the average framing energy denoted by AFE to extract features from the *Z* frames of each WT ECG subsignal:
  (3) $$\begin{array}{c} \left\{\;u_{q}\left(\;t\;\right)\;\right\}=\left\{\;u_{q1},u_{q2},\ldots ,u_{q1Z}\;\right\}, \end{array}$$
 where *Z* is the number of considered frames for the *q*th WT subsignal *u*_*q*_(*t*). The percentages of energy corresponding to the terminal nodes of the WP tree (*E*) for the *Z* frames of *u*_*q*_(*t*) are utilized to extract a wavelet subsignal feature vector as follows:
  (4) $$\begin{array}{c} {afe}_{q}=\frac{1}{Z}\overset{Z}{\underset{z=1}{\sum }}e\left(\;u_{qz}\left(\;t\;\right)\;\right), \end{array}$$
 where *e*(*u*_*qz*_(*t*)) is the percentage of energy of *u*_*qz*_(*t*). The feature vector of the whole given ECG signal is
  (5) $$\begin{array}{c} AFE=\left\{\;afe_{1},{afe}_{2},\ldots ,{afe}_{Q}\;\right\}. \end{array}$$

To calculate the percentages of energy, the following equation is used:

(6) ES=100∗∑n=1Nuqt2NN=sumC2,

where *S* is the signal and *C* is the wavelet decomposition vector [1].

(iii) The extracted features of wavelet average framing percentage energy will be added for classification.

**Figure 1:**
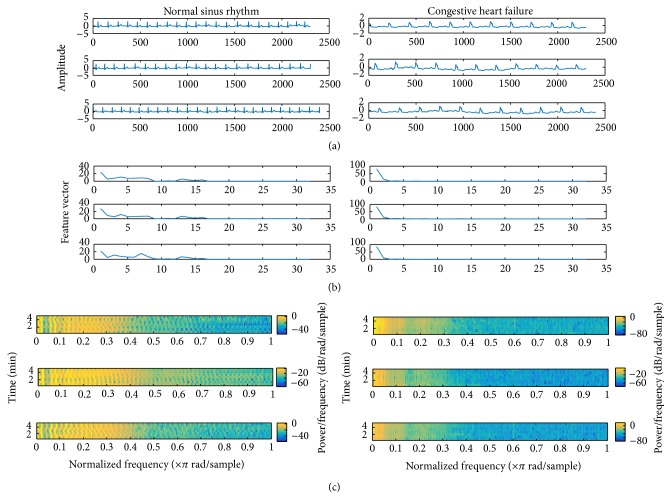
The WAFE and spectrogram for two ECG signal cases: NSR and CHF. (a) illustrates three cases of normal atrial rhythm (NSR) signals and three cases of CHF signals. (b) illustrates the same cases, this time by feature extraction vectors of the proposed method. It can be seen that the features have similar shapes for each distinct arrhythmia case. (c) Spectrogram of the two types.

The subjective evaluation of the proposed feature extraction method for the NSR and CHF classification for diagnosing tasks is shown in Figure 1, where Figure 1(a) shows three cases of normal atrial rhythm (NSR) signals and three cases of CHF signals.  Figure 1(b) illustrates the same cases but by the feature extraction vectors of percentages of energy corresponding to the terminal nodes of the WP. It can be seen that the features have similar morphology for a similar arrhythmia case.  Figure 1(c) shows spectrogram using a Short-Time Fourier Transform (STFT) of the two types. We can notice the distinctions. For more details, we can see a block diagram of the feature extraction method stages as follows:

 Preprocessing contains filtration from high-pass noise and baseline wandering. Normalization is also conducted to guarantee the same level of amplitudes.
 Signal decomposes into WPT tree at level five of *Q* number of subsignals.
 The percentages of energy corresponding to the terminal nodes of *Z* frames of each wavelet subsignal are calculated and then averaged over these frames.
 The feature vector of the whole given ECG signal is composed of each WPT subsignal average.

## 3. Classification by Confirmation Functions

Depending on the application, the universal area of arrhythmia recognition may be divided into two particular tasks: arrhythmia detection (identification) and arrhythmia confirmation (verification). In arrhythmia detection, the goal is to decide which one of a group of known arrhythmias best matches the input ECG sample.

Statistical analysis is used to examine the random data by calculating different statistical parameters. For this reason, two types of statistical moments may be used: (i) moment about zero such as mean square value, root mean square value, mean value, and correlation and (ii) moment about mean value, such as variance, covariance, and standard deviation. In the following experiment, statistics have been calculated for the proposed feature vectors of two cases of CHF and two cases of PEB, including standard deviation, median, max., and variance. The results are tabulated in Table 1. The results show that the statistical parameters of CHF1 and CHF2 are very close, which is similar for PEB1 and PEB2. The results are almost the same for all testing cases used in our paper.

In arrhythmia confirmation, the task is to decide from an ECG sample whether a patient's case diagnosis is the right one or not. In this work, we will take the CHF confirmation track. The basis for presenting our CHF recognition system is the WAFE method, which is used as a feature extraction method. More specifically, the feature vectors extracted from a person's ECG are modeled by WAFE to be given for classification (confirmation task). For a feature vector denoted by *x*, the model for the ECG of CHF is defined as the average vector calculated for fifteen different ECG signal segments (10 sec time duration) of a CHF case of Einthoven's standard leads. This average vector is used to present the CHF model termed in this paper as a hypothesized ECG arrhythmia to be compared to background (opposite) ECG arrhythmia that presents one of the other ECG arrhythmias. Background ECG arrhythmia is defined as the average vector calculated for fifteen ECG signals of other types, such as NSR, premature ectopic beat (PEB), and AF of many patients. This idea came from the single-speaker detection task [45]. It can be defined as a basic hypothesis test between *H*_0_ (*Y* is from the hypothesized arrhythmia) and *H*_1_ (*Y* is not from the hypothesized arrhythmia). The optimum test to choose between these two hypotheses is the following ratio test:

(7) $$\begin{array}{c} \frac{C\left(Y,H_{0}\right)}{C\left(Y,H_{1}\right)}=\left\{\begin{array}{cc} \geq \theta \cdots Accept & \\ <\theta \cdots Reject, & \end{array}\right. \end{array}$$

where *C*(*Y*, *H*_*i*_), *i* = 0, 1, is the confirmation function for the hypothesis *H*_*i*_ evaluated for the tested ECG segment *Y*.

In this paper, we propose three confirmation functions. The motivations behind using these confirmation methods are as follows: (1) they are very simple; (2) they could be built by very simple mathematics; (3) they do not need sophisticated computer programs; (4) the proposed confirmation methods are very speedy and do not require a training stage. The three methods are summarized as follows.

*(i) Percentage Root Mean Square Difference Score (PRDS)*. This is the most prominently used distortion measure for quality evaluation of reconstructed signals in ECG compression analysis. This confirmation function is based on the distance concept given by

(8) PRDSY,Modelx,b=100%∗∑n=1NWAFEY−WAFEMx,b2∑n=1NWAFEY2,

where WAFE_*Y*_ is the feature vector taken for tested ECG segment *Y* by WAFE method. WAFEM_*x*,*b*_ is CHF model (*x*) or background model (*b*) and *N* is the feature vector length. The decision threshold for CHF confirmation is determined by

(9) $$\begin{array}{c} \frac{PRDS\left(Y,{Model}_{x}\right)}{PRDS\left(Y,{Model}_{b}\right)}=\left\{\begin{array}{cc} <1\cdots Confirm & \\ \geq 1\cdots Nonconfirm. & \end{array}\right. \end{array}$$

**Figure 2:**
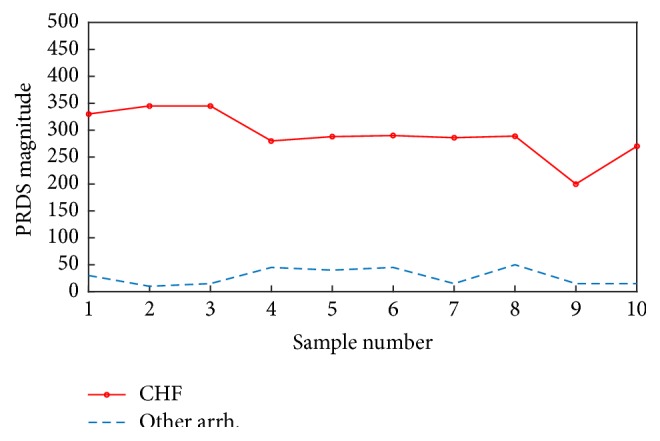
The ten PRDSs calculated between ten CHF signals and the CHF model and ten other PRDSs calculated between other arrhythmias (NSR and PEB).

PRDS is one of the most significant measures for determining the deformation happening in ECG signal after compression or filtration [36]. It calculates the difference error as a percentage score. This can be employed in determining the exact change (deformation) in the feature vector caused by different arrhythmias. For achieving real and better results by means of PRDS in CHF confirmation task by (9), several tricks should be taken into consideration:

- All ECG signals used in the experiment should be filtrated from high noise and from baseline wandering.
- All ECG signals used in the experiment should have the same sampling frequency and amplitude level.
- All ECG samples used in the experiment should be taken from one ECG lead type (it would be better to have the same lead, e.g., I, II, or V1 lead).
- The PRDS calculated between CHF signals and the CHF model must be at least five times less than that calculated between other arrhythmias and the CHF model. This is the most essential condition that justifies the using of (9) in confirmation.  Figure 2 illustrates a sequence of ten PRDSs calculated between ten CHF signals and CHF model and ten other PRDSs calculated between other arrhythmias (NSR and PEB) and CHF model. We can notice that the PRDSs of CHF signals are about 8 times less.

*(ii) Logarithmic Difference Tested Signal to Signal Model Ratio (LDSR)*. Consider the following:

(10) LDSRY,Modelx,b=Relog⁡∑n=1NWAFEY−WAFEMx2∑n=1NWAFEY−WAFEMb2.

The decision threshold for CHF confirmation is determined by

(11) $$\begin{array}{c} LDSR\left(\;Y,{Model}_{x,b}\;\right)=\left\{\begin{array}{cc} \leq zero\cdots Confirm & \\ >zero\cdots Nonconfirm. & \end{array}\right. \end{array}$$

LDSR calculates the power of the difference error as a ratio score. This can be employed in determining the difference between the testing features and signal model to the difference of the testing features and background model ratio in logarithmic scale. As we know, if the ratio is less than one, the logarithmic scale will be negative, leading to the classification of the testing features as CHF features. When the ratio is more than one, the logarithmic scale will be positive, and the testing features will be classified as a different type, like the opposite model. To achieve real and better results by means of LDSR in CHF confirmation task by (11), the first three tricks mentioned in the previous PRDS section should be taken into consideration.

*(iii) Correlation Coefficient Ratio (CCR)*. This function is denoted by correlation coefficient (CC) calculated between WAFE_*Y*_ and WAFE_*x*,*b*_ and the decision threshold is obtained by the following equation:

(12) CCRY,Modelx,bCCY,xCCY,b=≤1⋯Nonconfirm>1⋯confirm.

For more details, we can see a block diagram of classification method stages as follows:

 CHF model denoted by *x* is built by averaging of 15 feature extraction vectors calculated from CHF signals. The background model denoted by *b* is built from averaging of 15 feature extraction vectors calculated from NSR or PEB signals.
 Feature vector of the testing signal denoted by *Y* is calculated.
 Calculate the three confirmation methods, PRDS, LDSR, and CCR, between testing feature vector and the two models, CHF model and background model.
 Accept decision taking by the three thresholds: Th1 = (PRDS_*x*_/PRDS_*b*_) ≤ one, confirm; Th2 = LDSR ≤ zero, confirm; Th3 = (CC_*x*_/CC_*b*_) > one, confirm; otherwise, it is not CHF.

In this study, we used the wavelet ECG feature vectors, with three confirmation methods to be categorized. The motivations behind this choice are summarized as follows. (1) For wavelet, the essential properties of the arrhythmias, such as CHF or AF, are based on frequency and amplitude changes [1]. Therefore, the high irregularity in the heart rate is a major indicator of these arrhythmias so that the use of wavelet transform would help enormously in feature catching because of the possibility of the signal decomposing over several subbands of frequency. (2) For the confirmation methods, the feature vector is relatively not long enough that it would not affect the algorithm computational complexity. On the other hand, the possibility of working in an unsupervised and simple manner makes the algorithm perform online. This is easier for implementation and gives the ability to provide confidence in a decision based directly on the simple threshold [1]. Although this process does not affect the system performance, it will offer speedy processing and perform in a time efficient manner.

**Figure 3:**
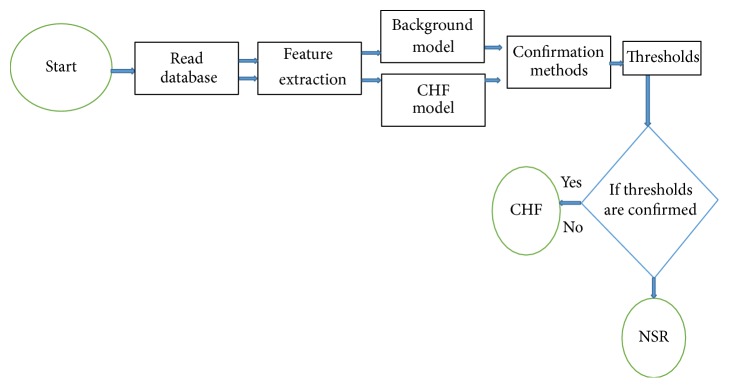
The classification algorithm flow chart.

Figure 3 illustrates the algorithm flow chart for the classification of the CHF and NSR, where a background model was built from NSR.

## 4. Results

One of the enormously popular ECG databases is the MIT-BIH Arrhythmia Database [46, 47] that contains 48 half-hour excerpts from two-channel ambulatory ECG recordings made at the BIH Arrhythmia Laboratory between 1975 and 1979. The congestive heart failure (CHF) type was obtained from the BIDMC congestive heart failure database [47], where 150 signals were taken for algorithm testing. The duration of the recordings is 10 hours, and they have two ECG signals of 250 Hz sampling frequency, with 12 bit resolution over a range of 10 mV. The original analog recordings were made at Boston's Beth Israel Hospital using ambulatory ECG recorders with a customary recording bandwidth of about 0.1 Hz to 40 Hz. For atrial fibrillation (AF) arrhythmias, we utilized the MIT-BIH atrial fibrillation database [46]. This database contains 25 long-term ECG recordings of patients with atrial fibrillation. Our investigation of confirmation system performance for AF arrhythmias was conducted via several experiments using 170 signals, each 10 seconds long and of about 12 beats (this standard is common for each ECG signal used in this study). The signals were taken from several records such as 04936 and 04015. The number of individual signals that were used for the algorithm investigation is 170, and they were AF-type signals. Normal sinus rhythm signals taken from the MIT-BIH normal sinus rhythm database were also used for algorithm testing. This database includes 18 long-term ECG recordings of subjects referred to the Arrhythmia Laboratory at the same hospital. Signals included in this database have had no significant arrhythmias; they include five men, aged 26 to 45, and 13 women, aged 20 to 50. 142 signals (type NSR) of 10 seconds were used.

The signals have the same recording specifications as in the MIT-BIH atrial fibrillation database. In addition, 150 premature ectopic beat (PEB) signals taken from the MIT-BIH normal sinus rhythm database (excluded because of the presence of occasional ectopic beats) were used for algorithm examination. CHF can be seen in all 12 leads. For classification purposes, any lead can be chosen, but the choice must be common for all selected signals. However, the same leads are selected for all the ECG signals used in algorithm testing. Mainly, Einthoven leads (I, II, and III) were used in the experiments to evaluate the classification process [1]. The recognition performance is accomplished as an average of three measures: sensitivity, specificity, and positive predictivity [23]. The Receiver Operating Characteristic (ROC) curve figure is also used for testing the method [48, 49].

In the method, we propose a study of the CHF recognition by WAFE in normal and noisy environments. In other words, the presented study may be considered as an investigation aiming to build a system that recognizes the CHF arrhythmias even with the noisy signals. The system is applied to a huge number of testing signals. The CHF confirmation track will be taken. We solve the problem using the general recognition method (feature extraction and then classification). This approach is based on a combination of percentage energy and WT to accomplish feature extraction of the arrhythmias obtained from normalized and interferences removed signals. The obtained feature extraction vector is utilized for classification by means of the proposed three confirmation methods. The decision of any tested sample is obtained depending on the confirmation, or not, of the three methods together. If the three methods confirm, the decision is “accepted,” but if one or more of these methods are not confirmed, the result will be rejected. The motivation behind using these confirmation methods is because they are very simple; they can be built using very simple mathematics and do not need sophisticated computer programs. The proposed confirmation methods are speedy and do not require a training stage.

Fifteen ECG patterns of CHF arrhythmia were utilized for CHF model building as well. The background models are built for each individual arrhythmia (NSR, AF, and PEB) to be used in the confirmation methods. 462 different arrhythmias, ECG segments (NSR, AF, and PEB), and 150 CHF ECG segments were used for algorithm investigation. The recognition performance was accomplished as 92.60% (when CHF and NSR were recognized, it means that the background model was built from 15 NSR signals) as an average of three measures (Table 2): (1)* sensitivity* (%) = (TP/(TP + FN)) × 100%, (2)* specificity* = (TN/(TN + FP)) × 100%, and (3)* positive predictivity* = (TP/(TP + FP)) × 100% (see Table 2), where TN is a true negative results number when the system identifies the testing signal as the background (opposite case: NSR, AF, or PEB). For example, NSR is a background, and TP is a true positive result when the system confirms the tested signal as CHF, in case the tested signal really belongs to CHF. Table 2 tabulates the recognition rate for the proposed method. The choice of the wavelet mother function type is extremely important and is dependent on the intended application. In our study we have investigated many wavelet functions and their corresponding recognition rate. Based on our investigation, we have chosen to use the wavelet function type Daubechies five (also known as db5) on the basis that it yields the best recognition rate. Therefore, it will be considered for the rest of our investigation.

**Figure 4:**
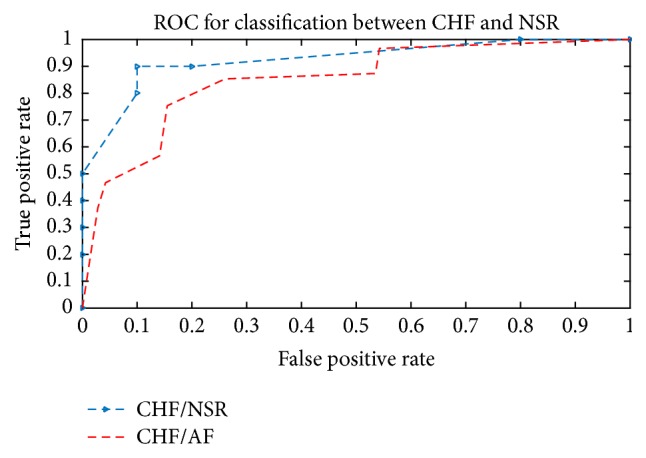
ROC curves for CHF and NSR recognition and CHF and AF recognition. The areas under the curve (AUC) were 0.9257 and 0.8549, respectively.

The Receiver Operating Characteristic (ROC) curve investigation is a common tool for evaluating the diagnostic or discrimination accuracy of abnormal cases from normal cases (or another type of abnormality). When we consider the results of a particular test in two cases, we will seldom find identical discrimination between the two groups. Generally speaking, the distribution of the test results will overlap. Therefore, some abnormal cases are correctly classified as positive (TP fraction) while others are classified as negative (FN fraction); similarly, some normal cases are correctly classified as negative (TN fraction) while some are classified as positive (FP fraction). Accordingly, parameters can be employed to determine the true and false positive rates: true positive rate (TPR) = TP/(TP + FN); false positive rate (FPR) = fallout = FP/(FP + TN) = FAR; false rejection rate (FRR) = FN/(TP + FN) = 1TPR; false acceptance rate (FAR) = FP/(FP + TN) = FPR. The ROC curve plots the TPR (sensitivity) against the FPR for a set of cut-off points. The curve illustrates a sensitivity/specificity pair with regard to a certain decision threshold. An experiment with excellent discrimination has a ROC curve that goes through the upper left corner. Therefore, the closer the curve to the upper left corner, the higher the accuracy of the test.  Figure 4 demonstrates three ROC curves: for CHF and NSR recognition and CHF and AF recognition. The ROC curve of the three tests passes through the upper left corner, which indicates that the tests are reliable. The areas under the curve (AUC) were 0.9257 and 0.8549, respectively.

## 5. Discussion

When we compare to other published works based on wavelet transforms with the proposed method WAFE, such as average PSD of DWT (WPAP) [23], Shannon entropy with wavelet packet (WPSE) [50], log-energy entropy with wavelet packet (WPLE) [44], and sure entropy with wavelet packet (WPSUE) [51], we achieve a higher success rate (92.60%), where WPLE has reached only 83.14% recognition rate. The results are tabulated in Table 3. The Gaussian noise is a random variable stream with a Gaussian distribution. For instance, the electromyographic signal (EMG) that can be found naturally in the measuring environment is one of the most common interferences, which is a Gaussian random variable. Therefore, a white Gaussian noise environment is investigated in Table 4.

In the following experiments, the feature extraction method with the confirmation methods was analyzed to expose the usefulness of the proposed system in noisy environments. The following experiment investigates the proposed method in terms of recognition rate in additive white Gaussian noise, denoted by AWGN, with 5 and 0 dB SNR. This can be concluded from interpretation of the results in Table 4, where the results of WAFE in a noisy environment are tabulated. It was found that the recognition rates (in case of 5 dB SNR) of WAFE have a very good rate (81.48%). The reason behind that is the wavelet transform's ability to analyze the signal by different frequency subbands, and then the features are taken from places where the noise might be filtered by the wavelet transform.

Many studies that tackled CHF discrimination appeared lastly in the literature and were used for comparison, such as bispectral analysis and genetic algorithm [52]. The results of sensitivity and specificity rates of 93.10% and 98.14%, respectively, were claimed. Asyali's method [53] extracts nine features that were fed to the Bayesian classifier for distinguishing CHF from NSR. The results of sensitivity and specificity rates of 81.82% and 98.08%, respectively, were claimed. Another method was proposed by Işler and Kuntalp [54], where statistical time domain, frequency domain, and wavelet entropy features were fed to a KNN classifier. The results of sensitivity and specificity rates of 84.75% and 83.33%, respectively, were achieved. These three studies used NSR records and CHF records from PhysioNet and used the leave-one-out cross-validation system to evaluate the performance. Based on our CHF database taken from BIDMC congestive heart failure database and NSR database taken from the MIT-BIH and our different way of training and testing, the results of sensitivity 92.10% and specificity 92.86% were achieved. Relatively speaking, our method could be highly competitive. However, the comparison will be more accurate if all methods are tested over the same conditions, such as database and training/testing systems. In Table 5, a comparison with published works was conducted. The results show that our proposed method is superior in terms of recognition rate.

## 6. Conclusions

In the presented research paper, a CHF arrhythmia system based on WAFE was studied in normal and noisy environments. The proposed method is an important step toward achieving an automatic and accurate diagnosis system in the absence of cardiologists. Our method is simple and avoided the use of compute intensive algorithms such as genetic algorithms (GA) or artificial neural networks (ANNs). Hence, it does not require very sophisticated hardware.

The benefit of WAFE is its capability of reducing the huge ECG data and improving the computing speed. At the beginning of the feature extraction, WT is applied with percentage energy by analyzing the spectral parameters over a multiresolution space. The feature vector is then extracted from the signal obtained from wavelet coefficients. An average framing process was applied. For classification, three proposed confirmation methods were applied. The benefits of these methods are that they are simple, speedy, and accurate. The recognition performance of this method was demonstrated on the real ECG signals of different types taken from popular databases. Four different ECG arrhythmias were used for system testing in the experiments. Three ROC curves, for CHF and NSR recognition, CHF and PEB recognition, and CHF and AF recognition, were demonstrated. The ROC curve of the three tests passes through the upper left corner, which indicates that the tests are reliable. As a comparison with other published methods, experimental results showed that both WAFE and WPLE are suitable for CHF feature extraction method even in a noisy environment, at 5 dB SNR level. However, in the case of WAFE, better performance was produced than in WPLE in terms of the recognition rate of the three confirmation methods. The reason behind this is the ability of the wavelet transformation method to analyze the signal using different frequency subbands. Features can then be extracted from places where the noise might be filtered using wavelet transform. The results of statistical analysis showed that both CHF and PEB cases have very close parameters. The results are almost the same for all test cases used in our paper. A comparison with published works showed that our proposed method is superior in terms of recognition rate. It was concluded that the proposed approach is potentially useful for the automatic classification of CHF.

## Figures and Tables

**Table 1:** Statistical analysis of the proposed feature.

Statistical parameter	CHF1	CHF2	PEB1	PEB2
Std.	0.0496	0.0497	0.0498	0.0498
Median	0.0002	0.0002	0.0001	0.0001
Max.	0.0993	0.0995	0.0998	0.0997
Var.	2.4564	2.4761	2.4845	2.4826

**Table 2:** Results of recognition rate for the proposed method.

Method is WAFE	TN/TP	FN/FP	Recognition rate
CHF with NSR	130/140	12/10	92.60%
CHF with PEB	123/130	27/20	85.16%
CHF with AF	145/131	25/19	86.57.01%

**Table 3:** Results of recognition rates for comparison.

Method	TN/TP	FN/FP	Recognition rate
WPAP	111/108	31/42	74.08%
WPSE	67/72	75/78	47.73%
WPLE	135/120	17/30	83.14%
WPSUE	100/102	42/48	68.80%

**Table 4:** Results of recognition rates in a noisy environment.

Method	TN/TP	FN/FP	Recognition rate
WPLE 0 dB	51/70	91/80	43.03%
WAFE 0 dB	62/82	80/68	50.89%
WPLE 5 dB	80/112	62/38	68.94%
WAFE 5 dB	110/125	32/25	81.48%

**Table 5:** A comparison of recognition rate of the proposed method with published works.

Method	Recognition rate
WAFE	92.60%
Asyali [55]	89.82%
CMIFS [56]	90.16
mRMR [57]	92.55
Işler's method (without GA) [54]	84.34

## References

[1] Daqrouq K., Alkhateeb A., Ajour M. N., Morfeq A. (2014). Neural network and wavelet average framing percentage energy for atrial fibrillation classification. *Computer Methods and Programs in Biomedicine*.

[2] Khorrami H., Moavenian M. (2010). A comparative study of DWT, CWT and DCT transformations in ECG arrhythmias classification. *Expert Systems with Applications*.

[3] Erol H. (1989). *Electrocardiography*.

[4] Arini P. D., Baglivo F. H., Martínez J. P., Laguna P. (2014). Evaluation of ventricular repolarization dispersion during acute myocardial ischemia: spatial and temporal ECG indices. *Medical and Biological Engineering and Computing*.

[5] Sandoe E., Sigurd B. (1991). *Arrhythmia—A Guide to Clinical Electrocardiology*.

[6] Zhang Z., Silva I., Wu D., Zheng J., Wu H., Wang W. (2014). Adaptive motion artefact reduction in respiration and ECG signals for wearable healthcare monitoring systems. *Medical and Biological Engineering and Computing*.

[7] Goldberger L., Goldberger E. (1977). *Clinical Electrocardiography*.

[8] Sideris D. A. *Primary Cardiology*.

[9] Thakor N. V., Zhu Y.-S., Pan K.-Y. (1990). Ventricular tachycardia and fibrillation detection by a sequential hypothesis testing algorithm. *IEEE Transactions on Biomedical Engineering*.

[10] Thakor N. V., Natarajan A., Tomaselli G. F. (1994). Multiway sequential hypothesis testing for tachyarrhythmia discrimination. *IEEE Transactions on Biomedical Engineering*.

[11] Clayton R. H., Murray A., Campbell R. W. F. (1993). Comparison of four techniques for recognition of ventricular fibrillation from the surface ECG. *Medical and Biological Engineering and Computing*.

[12] Clayton R. H., Murray A., Campbell R. W. F. (1994). Recognition of ventricular fibrillation using neural networks. *Medical and Biological Engineering and Computing*.

[13] Afonso V. X., Tompkins W. J. (1995). Detecting ventricular fibrillation. *IEEE Engineering in Medicine and Biology Magazine*.

[14] Khadra L., Al-Fahoum A. S., Al-Nashash H. (1997). Detection of life-threatening cardiac arrhythmias using the wavelet transformation. *Medical and Biological Engineering and Computing*.

[15] Zhang X.-S., Zhu Y.-S., Thakor N. V., Wang Z.-Z. (1999). Detecting ventricular tachycardia and fibrillation by complexity measure. *IEEE Transactions on Biomedical Engineering*.

[16] Wang Y., Zhu Y.-S., Thakor N. V., Xu Y.-H. (2001). A short-time multifractal approach for arrhythmia detection based on fuzzy neural network. *IEEE Transactions on Biomedical Engineering*.

[17] Tsipouras M. G., Fotiadis D. I. (2004). Automatic arrhythmia detection based on time and time-frequency analysis of heart rate variability. *Computer Methods and Programs in Biomedicine*.

[18] Teich M. C., Lowen S. B., Jost B. M., Vibe-Rheymer K., Heneghan C. (2001). Heart rate variability: measures and models. *Nonlinear Biomedical Signal Processing, Dynamic Analysis and Modeling*.

[19] Asyali M. H. Discrimination power of long-term heart rate variability measures.

[20] Stridth M., Sornmo L., Meuding C., Olsson B. Frequency trends of atrial fibrillation using the surface ECG.

[21] Tateno K., Glass L. A method for detection of atrial fibrillation using RR intervals.

[22] Güler I., Übeyli E. D. (2005). ECG beat classifier designed by combined neural network model. *Pattern Recognition*.

[23] Kara S., Okandan M. (2007). Atrial fibrillation classification with artificial neural networks. *Pattern Recognition*.

[24] Ouyang N., Ikeda M., Yamauchi K. (1997). Use of an artificial neural network to analyse an ECG with QS complex in V12 leads. *Medical & Biological Engineering & Computing*.

[25] Özbay Y., Ceylan R., Karlik B. (2006). A fuzzy clustering neural network architecture for classification of ECG arrhythmias. *Computers in Biology and Medicine*.

[26] Ceylan R., Özbay Y. (2007). Comparison of FCM, PCA and WT techniques for classification ECG arrhythmias using artificial neural network. *Expert Systems with Applications*.

[27] Froese T., Hadjiloucas S., Galvão R. K. H., Becerra V. M., Coelho C. J. (2006). Comparison of extrasystolic ECG signal classifiers using discrete wavelet transforms. *Pattern Recognition Letters*.

[28] Yu S.-N., Chen Y.-H. (2007). Electrocardiogram beat classification based on wavelet transformation and probabilistic neural network. *Pattern Recognition Letters*.

[29] Cervigón R., Sánchez C., Castells F., Blas J. M., Millet J. (2007). Wavelet analysis of electrocardiograms to characterize recurrent atrial fibrillation. *Journal of the Franklin Institute*.

[30] de Azevedo-Botter E., Nascimento C. L., Yoneyama T. I. (2001). A neural network with asymmetric basis functions for feature extraction of ECG P waves. *IEEE Transactions on Neural Networks*.

[31] Carlson J., Johansson R., Olsson S. B. (2001). Classification of electrocardiographic P-wave morphology. *IEEE Transactions on Biomedical Engineering*.

[32] Dierya A., Rowlandsa D., Cutmore T. R. H., James D. (2011). Automated ECG diagnostic P-wave analysis using wavelets. *Computer Methods and Programs in Biomedicine*.

[33] Dokur Z., Ölmez T., Yazgan E. (1999). Comparison of discrete wavelet and Fourier transforms for ECG beat classification. *Electronics Letters*.

[34] Vajarian K., Splinter R. (2006). *Biomedical Signal and Image Processing*.

[35] Daqrouq K., Abu-Isbeih I. N. Arrhythmia detection using wavelet transform.

[36] Daqrouq K., Abu-Isbeih I. N., Daoud O., Khalaf E. (2010). An investigation of speech enhancement using wavelet filtering method. *International Journal of Speech Technology*.

[37] Alfaouri M., Daqrouq K. (2008). Quality evaluation techniques of processing the ECG signal. *American Journal of Applied Sciences*.

[38] Alfaouri M., Daqrouq K. (2008). ECG signal denoising by wavelet transform thresholding. *American Journal of Applied Sciences*.

[39] Wu J.-D., Lin B.-F. (2009). Speaker identification using discrete wavelet packet transform technique with irregular decomposition. *Expert Systems with Applications*.

[40] Daqrouq K., Al Azzawi K. Y. (2012). Average framing linear prediction coding with wavelet transform for text independent speaker identification system. *Computers and Electrical Engineering*.

[41] Avci E., Hanbay D., Varol A. (2007). An expert discrete wavelet adaptive network based fuzzy inference system for digital modulation recognition. *Expert Systems with Applications*.

[42] Behroozmand R., Almasganj F. (2007). Optimal selection of wavelet-packet-based features using genetic algorithm in pathological assessment of patients' speech signal with unilateral vocal fold paralysis. *Computers in Biology and Medicine*.

[43] Sanchez C., Miller J., Rieta J. J., Castlls F., Rodenas J., Ruiz-Granell Ruiz V. (2002). Packet wavelet decomposition: an approach for atrial activity extraction. *Computer in Cardiology*.

[44] Qiao S., Zhou P. Wavelet and wavelet packet transform analysis in the ECG signals of Atrial Fibrillation.

[45] Reynolds D. A., Rose R. C. (1995). Robust text-independent speaker identification using Gaussian mixture speaker models. *IEEE Transactions on Speech and Audio Processing*.

[46] Goldberger A. L., Amaral L. A., Glass L. (2000). PhysioBank, PhysioToolkit, and PhysioNet: components of a new research resource for complex physiologic signals. *Circulation*.

[47] Moody G. B., Mark R. G. (2001). The impact of the MIT-BIH arrhythmia database. *IEEE Engineering in Medicine and Biology Magazine*.

[48] Metz C. E. (1978). Basic principles of ROC analysis. *Seminars in Nuclear Medicine*.

[49] Zweig M. H., Campbell G. (1993). Receiver-operating characteristic (ROC) plots: a fundamental evaluation tool in clinical medicine. *Clinical Chemistry*.

[50] Daqrouq K. (2011). Wavelet entropy and neural network for text-independent speaker identification. *Engineering Applications of Artificial Intelligence*.

[51] Avci D. (2009). An expert system for speaker identification using adaptive wavelet sure entropy. *Expert Systems with Applications*.

[52] Yu S.-N., Lee M.-Y. (2012). Bispectral analysis and genetic algorithm for congestive heart failure recognition based on heart rate variability. *Computers in Biology and Medicine*.

[53] Asyali M. H. Discrimination power of long-term heart rate variability measures.

[54] Işler Y., Kuntalp M. (2007). Combining classical HRV indices with wavelet entropy measures improves to performance in diagnosing congestive heart failure. *Computers in Biology and Medicine*.

[55] Köhler B.-U., Hennig C., Orglmeister R. (2002). The principles of software QRS detection. *IEEE Engineering in Medicine and Biology Magazine*.

[56] Cheng H., Qin Z., Qian W., Liu W. Conditional mutual information based feature selection.

[57] Peng H., Long F., Ding C. (2005). Feature selection based on mutual information criteria of max-dependency, max-relevance, and min-redundancy. *IEEE Transactions on Pattern Analysis and Machine Intelligence*.

